# Pathophysiological properties of CLIC3 chloride channel in human gastric cancer cells

**DOI:** 10.1186/s12576-020-00740-7

**Published:** 2020-02-17

**Authors:** Shunsuke Kawai, Takuto Fujii, Takahiro Shimizu, Kenta Sukegawa, Isaya Hashimoto, Tomoyuki Okumura, Takuya Nagata, Hideki Sakai, Tsutomu Fujii

**Affiliations:** 1 Department of Surgery and Sciences, Graduate School of Medicine and Pharmaceutical Sciences, University of Toyama, Toyama, 930-0194 Japan; 2 Department of Pharmaceutical Physiology, Graduate School of Medicine and Pharmaceutical Sciences, University of Toyama, 2630 Sugitani, Toyama, 930-0194 Japan

**Keywords:** Cell proliferation, Chloride channel, Chloride intracellular channel protein, Gastric cancer, Prognosis

## Abstract

Pathophysiological functions of chloride intracellular channel protein 3 (CLIC3) in human gastric cancer have been unclear. In the tissue microarray analysis using 107 gastric cancer specimens, CLIC3 expression was negatively correlated with pathological tumor depth, and the patients with lower expression of CLIC3 exhibited poorer prognosis. CLIC3 was expressed in the plasma membrane of cancer cells in the tissue. CLIC3 expression was also found in a human gastric cancer cell line (MKN7). In whole-cell patch-clamp recordings of the cells expressing CLIC3, NPPB-sensitive outwardly rectifying Cl^−^ currents were observed. Cell proliferation was significantly accelerated by knockdown of CLIC3 in MKN7 cells. On the other hand, the proliferation was attenuated by exogenous CLIC3 expression in human gastric cancer cells (KATOIII and NUGC-4) in which endogenous CLIC3 expression is negligible. Our results suggest that CLIC3 functions as a Cl^−^ channel in the plasma membrane of gastric cancer cells and that decreased expression of CLIC3 results in unfavorable prognosis of gastric cancer patients.

## Introduction

Gastric cancer is one of the most common malignant tumors in the abdominal region [1, 2]. Various treatments have been developed such as surgical resection, endoscopic therapy and chemotherapy [3–5]. However, the morbidity rate of gastric cancer has increased as the population ages [6]. Clarifying the mechanism of malignant traits is important to improve prognosis of gastric cancer.

So far, in gastric cancer, overexpression of several anion channels has been reported to be related to unfavorable prognosis of the patients: higher expression of chloride channel-3 (CLC-3) promotes cellular invasion in gastric cancer and predicts poor prognosis [7]. Overexpression of transmembrane protein 16A (TMEM16A), a Ca^2+^-activated Cl^−^ channel, also contributes to tumor invasion and poor prognosis of human gastric cancer [8]. Elevation of chloride intracellular channel 1 (CLIC1) is strongly correlated with lymph node metastasis, lymphatic invasion and pathological staging in gastric cancer [9].

Based on previous reports of anion channels described above, we tried to clarify pathophysiological functions of other anion channels in gastric cancer. The CLIC family is known to consist of six human members and to be a subgroup of the glutathione-S-transferase superfamily [10]. On the other hand, it has been reported that chloride intracellular channel 3 (CLIC3) plays roles as not only a soluble protein, but also an organellar membrane protein [10–12]. Moreover, CLIC3 is associated with poor prognosis in pancreatic cancer, breast cancer, ovarian cancer, and malignant pleural mesothelioma [13–17]. Taken together, we have raised two questions: (1) Does CLIC3 act as a Cl^−^ channel in the membrane?, and (2) Is CLIC3 expression associated with prognosis of gastric cancer?.

In the present study, we therefore investigated expression and function of CLIC3 protein in human gastric cancer cells.

## Materials and methods

### Chemicals

DMEM and RPMI1640 were obtained from FUJIFILM Wako Pure Chemical Industries (Osaka, Japan). Fetal bovine serum (FBS), anti-Xpress antibody (catalog number; 46-0528), SuperScript IV Reverse Transcriptase, Lipofectamine 3000, antibiotic–antimycotic and pcDNA4/His B vector were from Thermo Fisher Scientific (Waltham, MA, USA). pIRES2-AcGFP1 vector was from Takara Bio (Kusatsu, Japan). KOD-Plus DNA polymerase was from Toyobo (Osaka, Japan). SV Total RNA Isolation System was from Promega KK (Tokyo, Japan). Western Lightning ECL Pro was from PerkinElmer (Waltham, MA, USA). Anti-CLIC3 antibody (ab128941, catalog number; EPR8243), Alexa Fluor 488-conjugated anti-rabbit IgG and Alexa Fluor 568-conjugated anti-mouse IgG antibodies were from Abcam (Cambridge, UK). Anti-β-actin antibody (8H10D10, catalog number; 3700S) was from Cell Signaling Technology (Beverly, MA, USA). Horse-radish peroxidase-conjugated anti-rabbit and anti-mouse IgGs were from Millipore (Bedford, MA, USA). 5-Nitro-2-(3-phenylpropylamino)benzoic acid (NPPB) was from Research Biochemicals International (Natick, MA, USA). 4′,6-Diamidino-2-phenylindole (DAPI) was from Dojindo Laboratories (Kumamoto, Japan). Polyethylenimine Max (PEI-Max) and Differential Quik Stain kit were from Polysciences Inc. (Warrington, PA, USA). All other reagents were of molecular biological grade or of the highest grade of purity available.

### Cloning of CLIC3 gene

Total RNA was extracted from human colon cancer HT-29 cells using the SV Total RNA Isolation System, and then the cDNA was synthesized using the SuperScript IV Reverse Transcriptase according to manufacturer’s instructions. The entire CLIC3 gene (accession number; NM_004669) was amplified by PCR using KOD-Plus DNA polymerase and the following primers (sense primer: 5′-ATCGAATTCATGGCGGAGACCAAGCTCCAGCTG-3′, and anti-sense primer: 5′-TATTCTAGACTAGCGGGGGTGCACGGCGGGCC-3′). The PCR condition was 2 min at 94 °C, followed by 50 cycles of 15 s at 94 °C, 30 s at 60 °C, and 1 min at 68 °C. The PCR products were ligated into pcDNA4/His B (CLIC3-pcDNA4) vector equipped with Xpress-tag at the upstream of CLIC3 cDNA and pIRES2-AcGFP1 (CLIC3-pIRES2-AcGFP1) vector.

### Cell culture and transient transfection of CLIC3

Human embryonic kidney HEK293T cells were cultured in DMEM medium containing 10% FBS and 1% antibiotic–antimycotic at 37 °C in 5% CO_2_. Human gastric cancer cell lines MKN7, MKN74, MKN45, KATOIII, and NUGC-4 were cultured in RPMI1640 medium containing 10% FBS and 1% antibiotic–antimycotic at 37 °C in 5% CO_2_. CLIC3-pcDNA4 or CLIC3-pIRES2-AcGFP1 vector was transfected into HEK293T, KATOIII, and NUGC-4 cells using PEI-Max according to manufacturer’s instructions. In the CLIC3-pcDNA4 vector-transfected cells, the Xpress-attached CLIC3 protein was expressed.

CLIC3-siRNA (CGGACGUGCUGAAGGACUU) and negative control siRNA were purchased from Nippon Gene (Tokyo, Japan). Alexa 488-conjugated siRNA was obtained from Qiagen (Hilden, Germany). The siRNA (20 pmol) was transfected into MKN7 cells by using Lipofectamine 3000 in 24-well culture plate.

### Preparation of membrane fractions

Cultured cells were scraped and suspended in phosphate buffered saline (PBS) containing 5 mM EDTA. The suspension was centrifuged at 500×*g* for 3 min, and the pellet was washed with PBS. After washing, cells were incubated in low ionic salt buffer (0.5 mM MgCl_2_, 10 mM Tris–HCl, pH 7.4) on ice for 10 min. The cells were homogenized with Dounce homogenizer, and centrifuged at 500×*g* for 10 min. Then, the supernatant was centrifuged at 100,000×*g* for 90 min at 4 °C, and membrane fractions were prepared by resuspending the pellets in solution containing 250 mM sucrose and 5 mM Tris–HCl (pH 7.4).

### Immunocytochemical analysis

Cells were fixed with ice-cold methanol for 5 min at room temperature and then permeabilized with PBS containing 0.3% Triton X-100 and 0.1% bovine serum albumin (BSA) for 15 min at room temperature. Non-specific binding of antibodies was blocked with a solution containing 20 mM phosphate buffer (pH 7.4), 450 mM NaCl, 16.7% goat serum, and 0.3% Triton X-100. The cells were incubated with anti-CLIC3 (1:100) and anti-Xpress (1:100) antibodies overnight at 4 °C and then with Alexa Fluor 488-conjugated anti-rabbit IgG and Alexa Fluor 568-conjugated anti-mouse IgG antibodies (1:100) for 1 h at room temperature. DNA was visualized using DAPI (1:1,000). Immunofluorescence images were visualized by using a Zeiss LSM 780 laser scanning confocal microscope (Carl Zeiss, Oberkochen, Germany).

### Electrophysiological experiments

Whole-cell patch-clamp recordings were performed with an EPC-10 patch-clamp amplifier (HEKA Elektronik, Lambrecht, Germany). Patch master software (HEKA Elektronik) was used for command pulse control and data acquisition. Data were filtered at 2.9 kHz and digitized at 10 kHz. The acquired data were analyzed with WinASCD software (kindly provided by Prof. G. Droogmans) and Clampfit 10.6 software (Molecular Devices, Union City, CA, USA). Patch electrodes had a resistance of 2–4 MΩ when filled with pipette solution. The access resistance was electrically compensated by 70% to minimize voltage errors. Current–voltage relationships were made from currents measured by applying voltage step pulses of 500 ms from − 100 to + 100 mV in 20-mV increments or ramp pulses of 100 ms from − 100 to + 100 mV. Steady-state currents were averaged at 450–500 ms on the step pulses. The currents were normalized to the corresponding membrane capacitance. HEK293T cells overexpressing human CLIC3 (24 h after transfection) and MKN7 cells were used. The CLIC3-overexpressing HEK293T cells were identified by GFP fluorescence. The pipette solution contained 140 mM *N*-methyl-d-glucamine (NMDG), 14 mM HCl, 126 mM l-aspartic acid, 2 mM Na_2_ATP, 5 mM MgCl_2_, 10 mM HEPES, 1 mM EGTA (pH 7.3 with Tris). The standard bathing solution contained 145 mM NMDG, 145 mM HCl, 7 mM MgCl_2_, 2 mM CaCl_2_, 10 mM HEPES (pH 7.4 with Tris). The low Cl^−^ bathing solution contained 145 mM NMDG, 145 mM l-aspartic acid, 7 mM MgCl_2_, 2 mM CaCl_2_, 10 mM HEPES (pH 7.4 with Tris).

### Tissue microarray (TMA) analysis

Tumor specimens from 107 patients with gastric cancer who underwent a surgery at Toyama University Hospital from 2001 to 2008 were used (Additional file 1: Table S1 and Additional file 2: Fig. S1). The tumors were histologically diagnosed in the Department of Pathology, University of Toyama. The final stage of gastric cancer was confirmed pathologically according to the TNM Classification of Malignant Tumors, 8th edition. The ethics committee of the University of Toyama approved this study.

The TMA comprised 1.0-mm cores of tissues from the paraffin-embedded blocks of the surgical specimen described above. Paraffin blocks containing tumor tissue were selected, and the representative areas encompassing the tumors were marked directly on the blocks according to the corresponding hematoxylin–eosin (HE)-stained slides. The array block was cut into sections that were placed onto glass slides for HE staining and immunohistochemical analyses. The sections were treated with an anti-CLIC3 antibody at a dilution of 1:50.

The expression level of CLIC3 was evaluated from distribution of staining and its intensity in each section. Distribution of CLIC3 staining in the section was scored as 0 (0% of total area), 1 (1–50%) and 2 (51–100%). Intensity of CLIC3 staining was scored as 0 (absent), 1 (weak), 2 (moderate) and 3 (strong). We defined that the tissue section is “CLIC3-high” if sum of two scores was 3 and above. Two researchers who do not know clinicopathological information of patients independently evaluated the section.

### Western blotting

Membrane proteins (30 µg) were treated with 2% sodium dodecyl sulfate (SDS) plus 5% β-mercaptoethanol, and they were separated by electrophoresis on SDS–polyacrylamide gels, and transferred to PVDF membranes. Non-specific binding of antibodies was blocked with 5% non-fat milk. The membranes were incubated with anti-CLIC3 (1:1000), anti-Xpress (1:5000), and anti-β-actin antibodies (1:5000) overnight at 4 °C. HRP-conjugated anti-rabbit IgG and anti-mouse IgG antibodies were used as secondary antibodies (1:5000). The signals were visualized using Western Lightning ECL Pro. To quantify the chemiluminescence signals on the membranes, a FujiFilm’s LAS-4000 system and MultiGauge software (FujiFilm) were used.

Full images of the PVDF membranes obtained from Western blotting experiments are shown in Additional file 3: Fig. S2.

### Cell proliferation assay

In 24-well culture plate, KATOIII cells (9 × 10^3^ cells per well) and NUGC-4 cells (6 × 10^3^ cells per well) were transfected with CLIC3-pIRES2-AcGFP1 vector or pIRES2-AcGFP1 vector (mock). Total cell numbers in each well were counted on transfection (1st counting) and 48 h after transfection (2nd counting). We confirmed that cell density for gene transfection (1st counting) has been in similar values between mock transfection and CLIC3 gene transfection (KATOIII cells, 9 × 10^3^ cells; NUGC-4 cells, 6 × 10^3^ cells). Cell proliferation was defined as an increased cell numbers between 1st and 2nd countings.

The siRNA-transfected MKN7 cells were plated on 24-well culture plate. After 24-h culture, the cells were dissociated and replated on 24-well culture plate (1.5 × 10^4^ cells per well). Total cell numbers in each well were counted at 48 h after the replating.

### Transwell migration assay and invasion assay

The protocol for analyzing in vitro cell migration was based on a transwell migration assay (Boyden chamber assay). Falcon cell culture inserts (Corning, Corning, NY, USA) with porous membranes (pore-size 8 µm) were placed into a 24-well plate. The attractant (medium containing 10% FBS) was added to the lower chamber, and KATOIII or NUGC-4 cells (4.0 × 10^4^ cells) were suspended in serum-free medium and added to the upper chamber. The cells were incubated at 37 °C for 72 h. The migrated cells attached to the lower side of the membrane were fixed and stained using a Differential Quik Stain kit. Cells remaining on the upper side of membrane were removed with a cotton swab. The membranes containing the migrated cells were dried, and the cells were counted in three randomly selected fields (×200).

Cell invasion was assessed by using a Matrigel transwell invasion assay. Cell culture inserts with porous membranes (pore-size 8 μm) were coated overnight with 0.5 mg/ml Matrigel (Corning). The inserts were placed into a 24-well plate and the attractant (medium containing 10% FBS) was added to the lower chamber, and KATOIII cells (4.0 × 10^4^ cells) were suspended in serum-free medium and added to the upper chamber. The cells were incubated at 37 °C for 72 h. The invaded cells attached to the lower side of the membrane were fixed, stained and counted.

### Statistical analysis

Data are expressed as the mean ± SEM. Differences between groups were analyzed by Chi-square test, Fisher’s exact test and *t*-test. Overall survival and disease-specific survival were analyzed using the Kaplan–Meier method and the log-rank test. Statistical analysis was performed using JMP Pro 13.0.0 (SAS institute., Cary, NC, USA) and *p* values < 0.05 were considered to be significant.

## Results

### Expression of CLIC3 in human gastric cancer cells

**Fig. 1:**
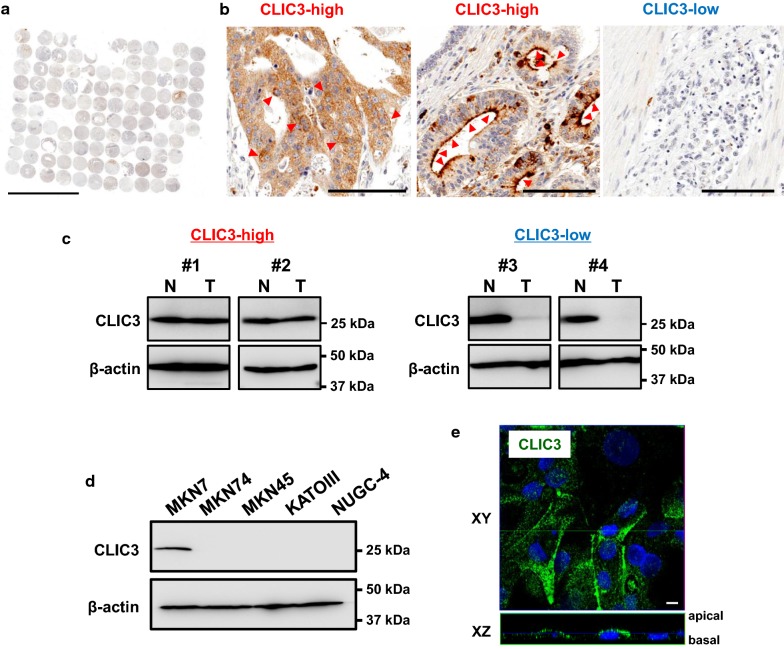
Expression of CLIC3 in human gastric cancer cells. **a** Tissue microarray (TMA) analysis using anti-CLIC3 antibody in the tumor of 107 patients with gastric cancer. Scale bar, 5 mm. **b** Enlarged images of the TMA samples judged as “CLIC3-high” (*left and middle*) and “CLIC3-low” (*right*). Red arrowheads in *left* and *middle* panels indicate expression of CLIC3 in apical side. Scale bars, 100 µm. **c** Representative images of Western blotting of gastric cancer tissues (T) and adjacent non-cancer tissues (N) in CLIC3-high (#1 and #2) and CLIC3-low (#3 and #4) samples. #1, 85F, non-solid type poorly differentiated adenocarcinoma, T4aN2M0, StageIIIA; #2, 58 M, signet-ring cell carcinoma, T4aN3aM0, StageIIIB; #3, 67 M, signet-ring cell carcinoma, T1bN0M0, StageIA, #4, 69 M, solid type poorly differentiated adenocarcinoma, T1bN0M0, StageIA. Clinicopathological explanation of specimens is given in the footnote of Table 1. Single band of CLIC3 was observed at 27 kDa. **d** Expression levels of CLIC3 in the membrane fractions of five gastric cancer cell lines. Expression of β-actin (45 kDa) was used as a loading control. **e** Immunocytochemistry of MKN7 cells using anti-CLIC3 antibody (green). Cell nucleuses were stained with DAPI (blue). XY and XZ images are shown. Scale bars, 10 µm

In a TMA of gastric cancer (107 specimens) treated with the anti-CLIC3 antibody, significant expression of CLIC3 protein (“CLIC3-high”; see Materials and methods) was found in 49 specimens (Fig. 1a, b, and Table 1). In the specimens judged as “CLIC3-high”, CLIC3 protein was localized in both the plasma membrane and intracellular compartment of cancer cells (Fig. 1b). In the CLIC3-high samples, expression level of CLIC3 in the cancer tissue was comparable to that of adjacent non-cancer tissue (Fig. 1c, left). In the “CLIC3-low” samples, however, expression level of CLIC3 in the cancer tissue was much lower than non-cancer tissue (Fig. 1b, c, right).

**Table 1:** Relationship between expression of CLIC3 and clinicopathological characteristics of patients with gastric cancer

	CLIC3-high (*n* = 49)	CLIC3-low (*n* = 58)	*p*-value
Gender			
Male	32	41	0.5516
Female	17	17	
Age			
≥ 65	35	39	0.6399
< 65	14	19	
pT			
T1a–T2	17	10	0.0381*
T3–T4b	32	48	
pN			
N0	15	17	0.8835
N1–3	34	41	
H			
H0	47	54	0.5234
H1	2	4	
P			
P0	44	55	0.3241
P1	5	3	
M			
M0	37	41	0.5754
M1	12	17	
CY			
CY0	43	48	0.4676
CY1	6	10	
pStage			
IA–IIB	26	22	0.1165
IIIA–IV	23	36	
Histology			
pap/tub1/tub2	17	30	0.0913
por1/por2/sig/muc	31	28	
ly			
−	8	7	0.5282
+	41	51	
v			
−	11	14	0.8369
+	38	44	

*pT* pathological tumor depth, *T1a* tumor invades lamina propria or muscularis mucosae, *T1b* tumor invades submucosa, *T2* tumor invades muscularis propria, *T3* tumor invades subserosa, *T4a* tumor perforates serosa, *T4b* tumor invades adjacent structures, *pN* pathological lymph node metastasis, *N0* no regional lymph node metastasis, *N1* metastasis in 1 to 2 regional lymph nodes, *N2* metastasis in 3 to 6 regional lymph nodes, *N3* metastasis in 7 or more regional lymph nodes, *H* liver metastasis;* H0* no liver metastasis, *H1* liver metastasis, *P* peritoneal dissemination, *P0* no peritoneal dissemination, *P1* peritoneal disseminationm, *M* distant metastasis, *M0* no distant metastasis, *M1* distant metastasis, *CY* peritoneal lavage cytology, *CY0 *no peritoneal lavage cytology, *CY1* peritoneal lavage cytology, *pStage* pathological stage (TNM stage), *StageIA* T1N0M0, *StageIB* T1N1M0 or T2N0M0, *StageIIA* T1N2M0 or T2N1M0 or T3N0M0, *StageIIB* T1N3aM0 or T2N2M0 or T3N1M0 or T4aN0M0, *StageIIIA* T2N3aM0 or T3N2M0 or T4aN1M0 or T4aN2M0 or T4bN0M0, *StageIIIB* T1N3bM0 or T2N3bM0 or T3N3aM0 or T4aN3aM0 or T4bN1M0 or T4bN2M0, *StageIIIC* T3N3bM0 or T4aN3bM0 or T4bN3aM0 or T4bN3bM0, *StageIV* anyTanyNM1. Histology, *pap* papillary adenocarcinoma, *tub1* well differentiated tubular adenocarcinoma, *tub2* moderately differentiated tubular adenocarcinoma, *por1* solid type poorly differentiated adenocarcinoma, *por2* non-solid type poorly differentiated adenocarcinoma, *sig* signet-ring cell carcinoma, *muc* mucinous adenocarcinoma, *ly* lymphatic invasion, *ly (−)* no lymphatic invasion, *ly ( +)* lymphatic invasion, *v* venous invasion, *v (−)* no venous invasion, *v ( +)* venous invasion

In Fig. 1d, expression levels of CLIC3 in membrane fractions of human gastric cancer cell lines were examined. CLIC3 was detected in MKN7 cells, while no significant signal of CLIC3 was observed in MKN74, MKN45, KATOIII, and NUGC-4 cells (Fig. 1d). In immunocytochemistry of MKN7 cells, endogenous CLIC3 was found to be expressed partly in the plasma membrane as well as intracellular compartment (Fig. 1e).

### Effects of CLIC3 expression on prognosis of gastric cancer patients

**Fig. 2:**
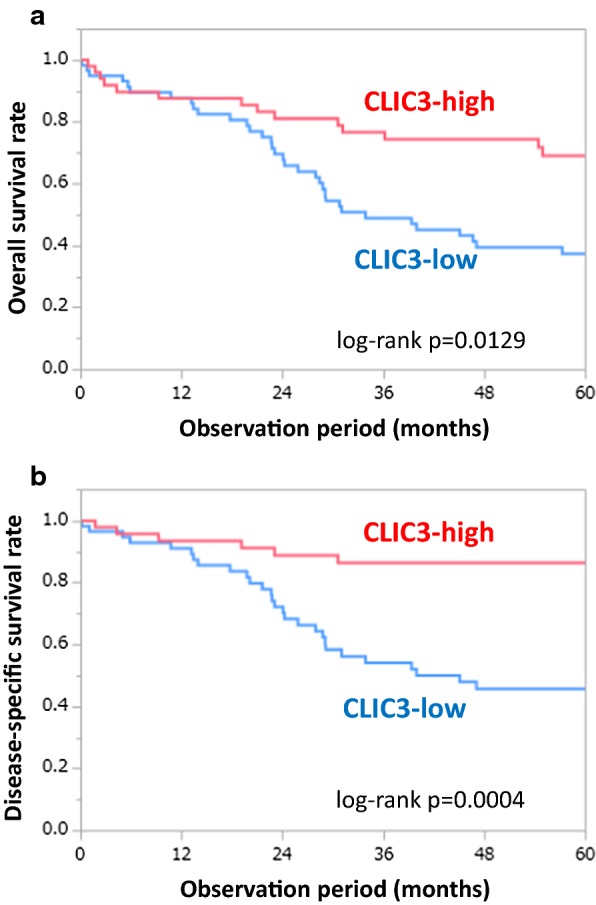
Effects of CLIC3 expression on prognosis of gastric cancer patients. **a** Correlation between expression level of CLIC3 and overall survival. **b** Disease-specific survival after surgery. They were analyzed by performing Kaplan–Meier survival analysis (log-rank test)

Correlation between expression level of CLIC3 and various prognostic factors is summarized in Table 1. Unexpectedly, expression level of CLIC3 was negatively correlated with pathological tumor depth: that is, the CLIC3-low samples showed deeper depth compared to the CLIC3-high samples. On the other hand, CLIC3 expression was not significantly related to gender, age, lymph node metastasis, liver metastasis, peritoneal dissemination, distant metastasis, peritoneal lavage cytology, pathological stage, histology, lymphatic invasion, and venous invasion (Table 1). Corresponding to above results, Kaplan–Meier survival curves showed that expression level of CLIC3 was negatively correlated with overall survival (Fig. 2a) and disease-specific survival (Fig. 2b): that is, the CLIC3-low patients showed poorer survival rate compared to the CLIC3-high patients. These results suggest that decreased expression of CLIC3 in gastric cancer may result in poor prognosis of the patients.

### Electrophysiology in the CLIC3-expressing cells

**Fig. 3:**
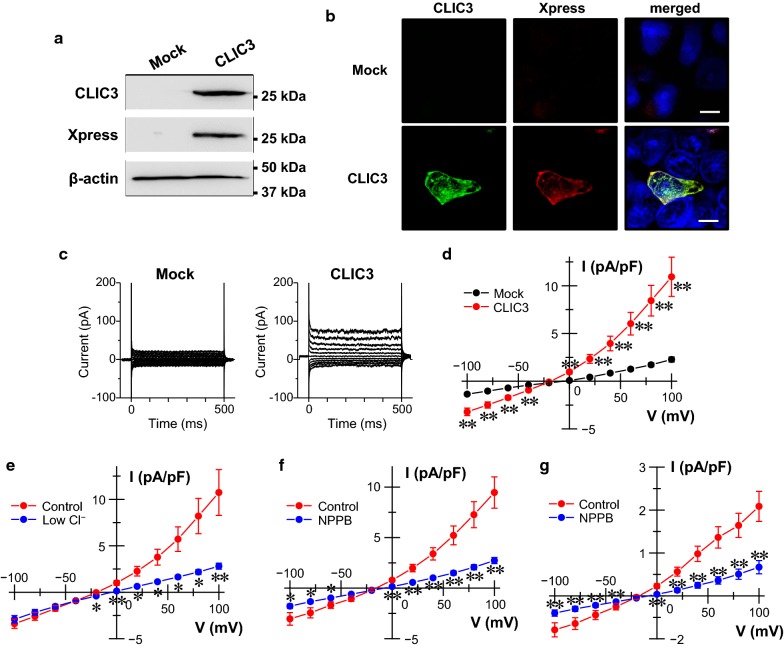
Electrophysiology in the CLIC3-expressing cells. **a** Expression of CLIC3 in the membrane fractions of mock (pcDNA4/His B vector)- and CLIC3 (CLIC3-pcDNA4 vector)-transfected HEK293T cells. Anti-CLIC3 and anti-Xpress antibodies were used to detect CLIC3 protein. **b** Immunocytochemistry of the mock (pcDNA4/His B vector)- and CLIC3 (CLIC3-pcDNA4 vector)-transfected HEK293T cells using anti-CLIC3 antibody (green; *left*) and anti-Xpress antibody (red; *middle*). Merged image is also shown (yellow; *right*). Cell nuclei was stained with DAPI (blue; *right*). Scale bars, 10 µm. **c** Representative traces of whole-cell currents obtained from mock (pIRES2-AcGFP1 vector)- and CLIC3 (CLIC3-pIRES2-AcGFP1 vector)-transfected HEK293T cells. **d** Current–voltage relationships of mock-transfected cells (black) and CLIC3-transfected cells (red). Each data point represents the means ± SEM of 10 and 15 experiments, respectively. **p < 0.01. **e** Current–voltage relationships of CLIC3-transfected cells exposed to standard bathing solution (control: red) and the low Cl^−^ bathing solution (blue). Each data point represents the means ± SEM of 11 experiments. *p < 0.05, **p < 0.01. **f** Current–voltage relationships of CLIC3-transfected cells in the absence (red) and presence (blue) of 100 µM NPPB. Each data point represents the means ± SEM of 10 experiments. *p < 0.05, **p < 0.01. **g** Current–voltage relationships of gastric cancer MKN7 cells endogenous expressing CLIC3 in the absence (red) and presence (blue) of 100 µM NPPB. Each data point represents the means ± SEM of 9 experiments. **p < 0.01

To clarify electrophysiological properties of CLIC3, it was exogenously expressed in HEK293T cells. A single band of CLIC3 was found in membrane samples of CLIC3 (CLIC3-pcDNA4 vector)-expressing cells, but not of empty vector-transfected (mock) cells by using anti-CLIC3 and anti-Xpress antibodies (Fig. 3a). In immunocytochemistry of the CLIC3-expressing HEK293T cells, CLIC3 was expressed partly in the plasma membrane as well as intracellular compartments (Fig. 3b). In whole-cell patch-clamp recordings, a significant outwardly rectifying Cl^−^ current was observed in CLIC3-expressing cells, but not in mock cells (Fig. 3c, d). As expected, the reversal potential of the current was positively shifted by a decrease in extracellular Cl^−^ concentration (Fig. 3e). The reversal potentials of the currents obtained by applying ramp pulses from − 100 mV to 100 mV under control and low Cl^−^ conditions were − 26.6 ± 4.2 and − 3.7 ± 2.1 mV (*n* = 3), respectively (Additional file 4: Fig. S3). NPPB, a Cl^−^ channel blocker, significantly decreased the currents (Fig. 3f). In gastric cancer MKN7 cells endogenously expressing CLIC3, similar Cl^−^ currents were observed (Fig. 3g). Expectedly, the currents were sensitive to NPPB. These results suggest that exogenous and endogenous CLIC3 proteins function as outwardly rectifying Cl^−^ channels in the plasma membrane.

### Knockdown of CLIC3 accelerated cell proliferation in MKN7 cells

**Fig. 4:**
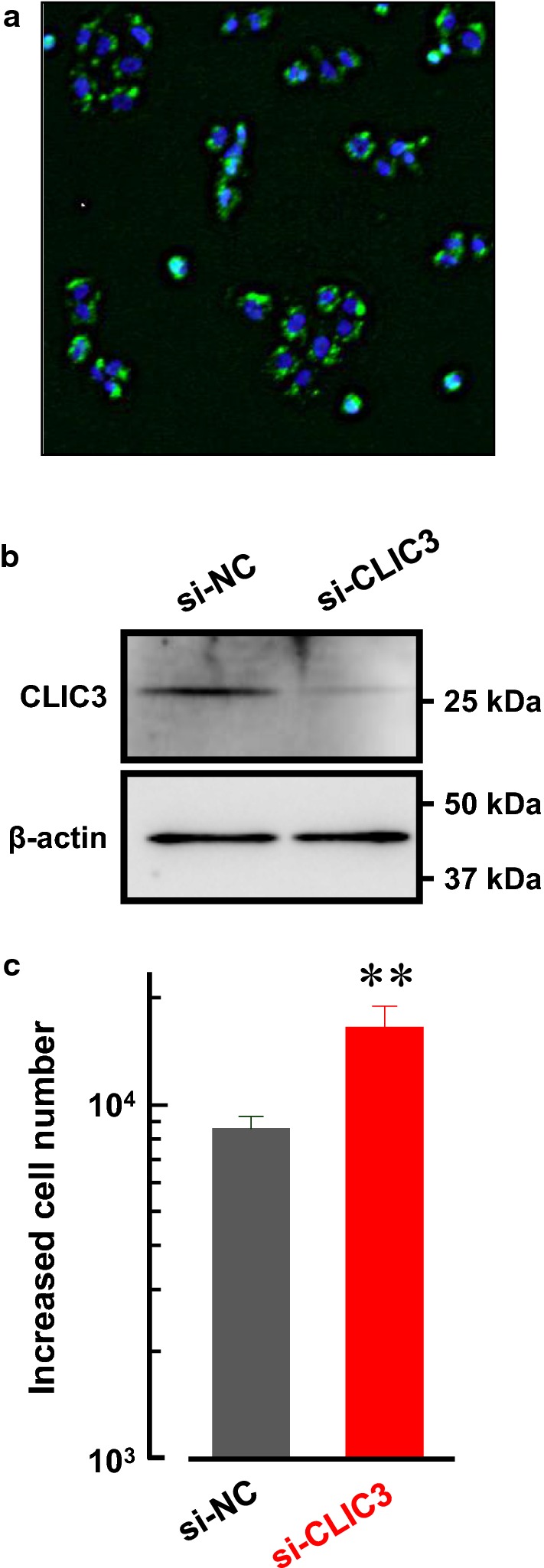
Knockdown of CLIC3 accelerated cell proliferation in MKN7 cells. **a** Transfection of Alexa 488-conjugated siRNA in MKN7 cells. **b** Expression level of CLIC3 in MKN7 cells transfected with negative control siRNA (si-NC) and CLIC3 siRNA (si-CLIC3). Anti-CLIC3 antibody was used to detect CLIC3 protein. Expression of β-actin (45 kDa) was used as a loading control. **c** Effects of CLIC3 expression on cell proliferation of MKN7 cells. Increased cell numbers at 48 h after replating are shown. Five independent experiments were performed. The bars represent the means ± SEM. **p < 0.01

Expression level of CLIC3 was found to be negatively correlated with pathological tumor depth (Table 1). Since tumor proliferation is associated with tumor depth, we examined effects of CLIC3 expression on cancer cell proliferation. CLIC3 was knocked down in MKN7 cells by using siRNA for CLIC3. Transfection efficiency was evaluated with fluorescence of Alexa 488-conjugated siRNA. Almost all the cells showed significant fluorescence (Fig. 4a). The expression level of CLIC3 protein was confirmed to be decreased dramatically in CLIC3 siRNA-transfected MKN7 cells compared with negative control siRNA-transfected cells (Fig. 4b). Interestingly, the increased cell number in CLIC3 siRNA-transfected MKN7 cells was significantly greater than that in negative control siRNA-transfected cells (Fig. 4c).

### Overexpression of CLIC3 attenuated cell proliferation in human gastric cancer KATOIII and NUGC-4 cells

**Fig. 5:**
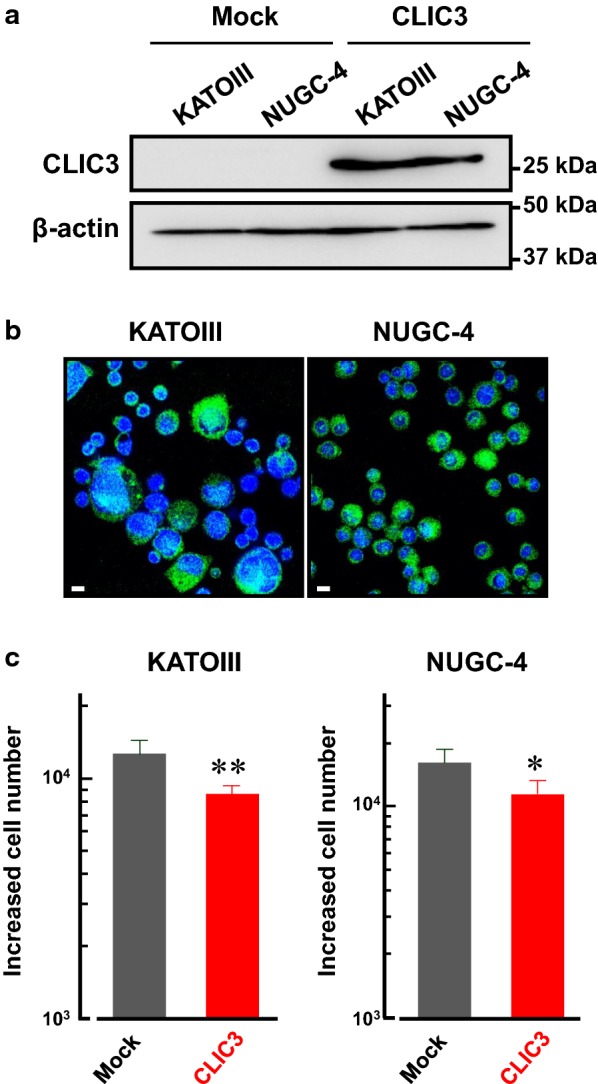
Overexpression of CLIC3 attenuated cell proliferation in KATOIII and NUGC-4 cells. **a** Expression level of CLIC3 in the membrane fractions of mock- and CLIC3-transfected KATOIII and NUGC-4 cells. Anti-CLIC3 antibody was used to detect CLIC3 protein (27 kDa). **b** Immunocytochemistry of KATOIII (*left*) and NUGC-4 cells (*right*) transfected with CLIC3 using anti-CLIC3 antibody (green). Cell nucleuses were stained with DAPI (blue). Scale bars, 10 μm. **c** Effects of CLIC3 expression on cell proliferation of KATOIII (*left*) and NUGC-4 cells (*right*). Increased cell numbers between 1st and 2nd countings (see Methods) are shown. Experiments were performed in triplicate. The bars represent the means ± SEM. *p < 0.05, **p < 0.01

Next, CLIC3 was overexpressed in KATOIII and NUGC-4 cells in which endogenous expression of CLIC3 is negligible (Fig. 5a). After transfection, almost all the cells showed significant fluorescence of CLIC3, indicating high efficiency of the transfection (Fig. 5b). The increased cell number of KATOIII and NUGC-4 cells expressing CLIC3 was significantly lower than their mock cells (Fig. 5c).

### No effects of CLIC3 overexpression on migration and invasion of KATOIII cells

**Fig. 6:**
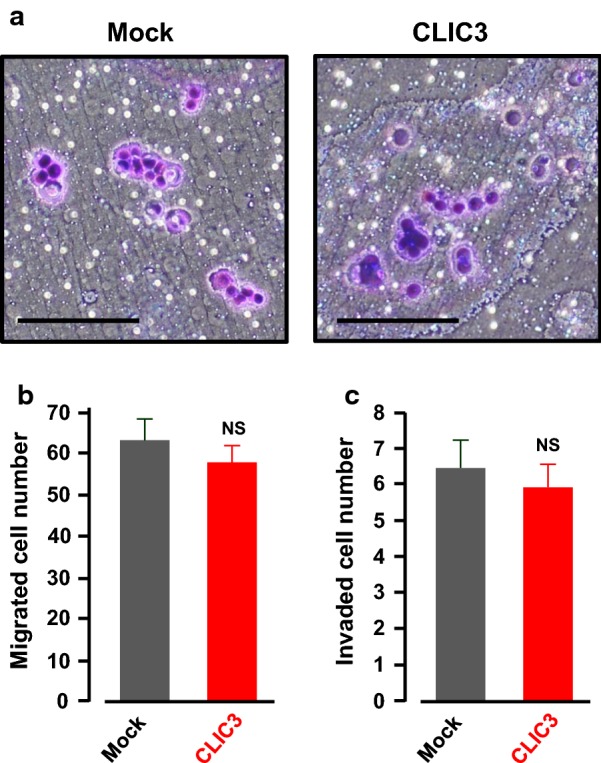
Transwell migration and invasion assay. **a** Morphology of mock- and CLIC3-transfected KATOIII cells. The migrating cells were stained with violet color. Scale bar, 100 µm. **b** Effects of CLIC3 expression on migration of the KATOIII cells. Migrated cell number in three different fields (each field = 400 × 400 µm) were counted in the experiment and the values were averaged. Three independent experiments were performed. The bars represent the means ± SEM. NS, not significant (*p* > 0.05). **c** Effects of CLIC3 expression on invasion of the KATOIII cells. Invaded cell number in three different fields (each field = 800 × 800 µm) were counted in the experiment and the values were averaged. Nine independent experiments were performed. The bars represent the means ± SEM. NS, not significant (*p* > 0.05)

Next we investigated abilities of migration and invasion of gastric cancer KATOIII cells transfected with an empty vector (mock) or a CLIC3 expression vector using a transwell (Fig. 6a). Consistent with results in Table 1, overexpression of CLIC3 had no significant effects on migration (Fig. 6b) and invasion (Fig. 6c) of the gastric cancer cells.

## Discussion

CLIC3 protein behaves as not only a soluble protein, but also an organellar membrane protein [10–12]. As a soluble protein, secreted CLIC3 promotes angiogenesis and invasion of ovarian cancer cells and breast cancer cells both in vivo and in 3D cell culture models by reducing transglutaminase-2 [16]. As an organellar membrane protein, CLIC3 in late endosome and lysosome promotes migration and invasion of pancreatic ductal adenocarcinoma by recycling integrins [13], and it also dictates invasion and metastasis of breast cancer by controlling recycling of late endosomal membrane-type matrix metalloproteinase-1 (MT1-MMP) [15]. These reports suggest that both soluble and organellar CLIC3 is associated with poor prognosis in cancers.

In the present study, the TMA analysis of patients with gastric cancer who underwent a surgery showed that expression level of CLIC3 was negatively correlated with overall survival and disease-specific survival (Fig. 2). It is noted that the relationship between expression level of CLIC3 and prognosis were opposite to the previous reports [13–17], although the types of cancer are different. In fact, expression level of CLIC3 was negatively correlated with pathological tumor depth (Table 1). Generally, tumor depth is associated with the proliferative capacity [18]. Our present results showed that cell proliferation was significantly enhanced by knockdown of CLIC3 in MKN7 cells, and that the proliferation was inhibited by exogenous CLIC3 expression in KATOIII and NUGC-4 cells in which endogenous CLIC3 expression is negligible (Figs. 1 and 5). These results suggest that decreased expression of CLIC3 stimulates their malignant potential in gastric cancer. On the other hand, metastatic potential is generally associated with the invasive and migratory capacity [19, 20]. In the present study, however, migration and invasion of cancer cells are not affected by exogenous CLIC3 expression in the gastric cancer cells.

Here, we also found that CLIC3 was partially expressed in the plasma membrane of gastric cancer cells in CLIC3-high patients and MKN7 cells. To our knowledge, it is the first report showing that CLIC3 is localized in the plasma membrane of cancer cells in human tissues. In fact, our patch-clamp experiments showed that both exogenous and endogenous CLIC3 function as outwardly rectifying Cl^−^ channels. In the present study, CLIC3 currents exhibited the reversal potentials at around − 27 mV. This value is shallower than the estimated equilibrium potential for Cl^−^ of − 48 mV. If it is assumed that aspartate ion partially permeates through CLIC3, the permeability of aspartate ion to Cl^−^ was calculated to be 0.34 ± 0.07 (*n* = 3) based on the reversal potential shift obtained from the ramp pulse experiments (Additional file 4: Fig. S3).

The activities of Cl^−^ channels in the plasma membrane are responsible for intracellular Cl^−^ homeostasis. So far, a close association between intracellular Cl^−^ concentration ([Cl^−^]_*i*_) and cancer cell proliferation has been reported. Gastric cancer MKN28 cells cultured in a low Cl^−^ medium exhibit a decrease in [Cl^−^]_*i*_ and reduced cell proliferation with G0/G1 arrest [21]. Interestingly, [Cl^−^]_*i*_ affects the anti-cancer activity of paclitaxel, a microtubule-targeted chemotherapeutic drug, in MKN28 cells [22]. A decrease in [Cl^−^]_*i*_ also attenuates cell proliferation of prostate cancer PC3 cells [23]. Changes in expression level of CLIC3 in the plasma membrane may disrupt intracellular Cl^−^ homeostasis in gastric cancer cells, resulting in the enhancement of cancer cell growth. Thus, the plasma membrane expression of gastric CLIC3 may explain why CLIC3 function in gastric cancers is different from other cancers. Future study is necessary to clarify the mechanism of regulation of CLIC3 expression in gastric cancer cells.

## Conclusions

Our study clarifies that CLIC3 has a channel activity in the plasma membrane of gastric cancer cells, and that decreased expression of CLIC3 results in unfavorable prognosis of gastric cancer patients via stimulation of the cancer cell proliferation. These findings suggest that pathophysiological role of CLIC3 in plasma membrane is different from that in cytosol and organellar membrane of the cancer cells.

## Supplementary information

**Additional file 1: Table S1.** Expression of CLIC3 and clinicopathological characteristics patients with gastric cancer.

**Additional file 2: Fig. S1.** Enlarged image of tissue microarray analysis (Fig. 1a) and the specimen numbers.

**Additional file 3: Fig. S2.** Full images of Western blotting shown in Fig. 1c (a), Figs. 1d and 3a (b) and Figs. 4b and 5a (c).

**Additional file 4: Fig. S3.** Representative current-voltage relationships of CLIC3 currents recorded with ramp pulses (100 ms) from − 100 mV to + 100 mV in the CLIC3-expressing HEK293T cells exposed to standard bathing solution (control: red) and the low Cl^−^ bathing solution (blue).

## Data Availability

Not applicable.

## Abbreviations

CLIC: Chloride intracellular channel
DAPI: 4′,6-Diamidino-2-phenylindole
FBS: Fetal bovine serum
[Cl^−^]_*i*_: Intracellular Cl^−^ concentration
HE: Hematoxylin–eosin
NPPB: 5-Nitro-2-(3-phenylpropylamino)benzoic acid
PBS: Phosphate buffered saline
PEI-Max: Polyethylenimine Max
TMA: Tissue microarray
