# Supplementary material for: Pathophysiological properties of CLIC3 chloride channel in human gastric cancer cells
Source: J Physiol Sci. 2020 Feb 17;70:15. doi: 10.1186/s12576-020-00740-7 (PMC7026216; doi:10.1186/s12576-020-00740-7)
Supplement: Supplementary file 1 — Additional file 1: Table S1. Expression of CLIC3 and clinicopathological characteristics patients with gastric cancer. [file 12576_2020_740_MOESM1_ESM.pdf]

Additional file 1: Table S1. Expression of CLIC3 and clinicopathological characteristics of patients with gastric cancer.

No	CLIC3	gender	age	pT	pN	H	P	M	CY	pStage	histology	ly	v	DS	IS	sum
1	high	m	65	2	0	0	0	0	0	1b	tub1>tub2	-	-	1	2	3
2	high	m	74	4a	3	0	0	0	0	3c	tub2>pap	+	+	1	2	3
3	high	m	82	2	0	0	0	0	0	1b	sig	+	+	1	2	3
4	low	m	74	4a	0	1	0	1	0	4	tub1	+	+	1	1	2
5	low	m	74	1b	2	0	0	0	0	2a	tub1	+	-	1	1	2
6	low	m	58	2	0	0	0	0	0	1b	tub2>por	-	-	1	1	2
7	low	m	86	4a	3	0	0	0	0	3c	por2	+	+	1	1	2
8	low	m	74	3	2	0	0	0	0	3a	tub2	+	+	1	1	2
9	low	m	50	4b	2	0	1	1	0	4	por2	+	+	1	1	2
10	low	f	58	4a	1	0	0	0	0	3a	por2	+	+	0	0	0
11	low	f	37	4a	3	0	0	1	0	4	por2>sig	+	+	1	1	2
12	low	m	71	4a	2	0	0	0	0	3b	tub2	+	+	0	0	0
13	high	m	82	4a	3	1	0	1	0	4	por1	+	+	2	1	3
14	low	f	72	3	3	0	0	0	0	3b	tub1>por2	+	+	0	0	0
15	low	m	64	1b	1	0	0	0	0	1b	tub2>por2	-	-	1	1	2
16	low	m	60	4a	1	0	0	1	1	4	muc	+	-	0	0	0
17	high	f	52	4a	3	0	1	1	0	4	por2	+	+	1	2	3
18	low	f	45	2	1	0	0	0	0	2a	por2	+	+	1	1	2
19	low	f	49	4b	3	0	0	1	0	4	por2>tub2	+	-	1	1	2
20	high	m	73	4a	3	0	1	1	0	4	por	+	+	2	1	3
21	high	m	67	4b	3	0	0	0	0	3c	muc	+	+	1	2	3
22	low	f	78	4a	3	0	0	0	0	4	pap>tub2	+	+	0	0	0
23	high	m	34	1b	3	0	0	0	0	2b	sig>por2	+	-	1	2	3
24	high	m	73	3	3	0	0	0	0	3b	tub2	+	+	1	2	3
25	high	m	73	3	0	0	0	0	0	2a	ecc	+	+	2	1	3
27	low	m	44	4a	0	0	0	0	0	4	por2>sig	+	+	1	1	2
28	high	f	79	3	0	0	0	0	0	2a	tub2>por1	+	+	2	1	3
29	high	m	70	1b	1	0	0	0	0	1b	pap-tub2	-	+	2	1	3
30	high	f	59	1b	1	0	0	0	0	1b	pap-tub1	+	+	2	1	3
31	low	m	83	3	2	0	0	0	0	3a	tub2-pap	+	+	0	0	0
32	low	m	84	4a	3	0	1	1	0	4	pap	+	+	1	1	2
33	low	m	47	3	0	0	0	0	0	2a	por1-sig	-	-	1	1	2
34	low	m	69	3	3	1	0	1	0	4	tub2>tub1	+	+	0	0	0
35	high	m	66	3	3	0	0	0	0	3b	sig	+	+	1	2	3

37	high	f	72	3	1	1	0	1	0	4	sig-por2	+	+	1	2	3
38	high	m	55	4a	3	0	0	0	0	3c	por2	+	+	1	2	3
39	high	m	74	3	3	0	0	0	0	3b	sig	+	+	1	2	3
40	low	m	68	3	2	0	0	0	0	3a	por2	+	+	1	1	2
42	low	m	74	4a	3	0	0	0	0	3c	por2>sig	+	+	1	1	2
43	high	m	75	2	1	0	0	0	0	2a	sig	+	-	1	2	3
44	high	m	76	3	0	0	0	0	0	2a	por1>tub2	-	+	2	2	4
45	low	f	59	2	2	0	0	0	0	2b	por2	+	-	1	1	2
46	high	m	67	1b	2	0	0	0	0	2a	tub2>pap	+	+	1	2	3
47	high	m	55	4a	1	0	0	0	0	3a	por2	+	+	1	2	3
48	high	f	65	2	0	0	0	0	0	1b	por2	-	+	1	2	3
49	low	m	69	4b	0	0	0	0	0	3c	por1	+	+	1	1	2
50	low	m	78	4a	3	0	0	0	0	3c	por	+	+	1	1	2
53	low	m	68	4a	3	0	0	1	1	4	por-sig	+	+	1	1	2
54	low	m	69	4a	3	0	0	0	0	3c	por1	+	+	1	1	2
55	high	f	85	1b	2	0	0	0	0	2a	tub2	+	+	1	2	3
56	low	m	65	4a	0	0	0	0	0	2b	tub2	+	+	1	1	2
57	low	f	76	2	0	0	0	0	0	1b	tub2>tub1	-	+	0	0	0
58	high	f	48	4a	0	0	0	0	0	2b	por2	+	+	1	2	3
59	high	m	81	3	3	0	0	0	0	3b	tub2	+	+	1	2	3
60	low	m	74	3	0	0	0	0	0	2a	tub2	-	-	1	1	2
61	low	m	62	3	0	0	0	0	0	2a	pap	-	+	0	0	0
63	low	m	70	4a	2	0	0	0	0	3b	por2	+	+	1	1	2
64	high	m	86	4a	0	0	0	0	0	2b	por2	-	-	1	2	3
65	low	m	73	3	1	0	0	1	1	4	tub2	+	+	1	1	2
66	high	m	50	3	3	0	1	1	1	4	por2	+	+	1	2	3
67	high	f	67	2	0	0	0	0	0	1b	por1	-	-	2	2	4
68	high	m	68	2	2	0	0	0	0	2b	tub1	+	+	1	2	3
69	high	f	67	4a	1	0	0	0	0	3a	por2	+	-	1	2	3
70	high	f	69	1b	1	0	0	0	0	1b	tub2	+	-	1	2	3
71	high	f	85	4a	2	0	0	1	1	4	sig	+	+	1	2	3
72	low	f	73	4a	1	0	0	1	1	4	tub2	+	-	1	1	2
73	low	m	76	3	2	0	0	0	0	3a	pap	+	+	1	1	2
74	high	m	71	3	3	0	0	0	0	3b	sig	+	+	1	2	3
75	low	m	61	4a	0	0	0	0	0	2b	tub2	+	+	1	1	2
76	low	f	77	4a	3	1	0	1	1	4	por1	+	+	0	0	0
77	low	m	72	4a	1	0	0	1	0	4	tub2	+	+	0	0	0
78	low	f	70	3	1	0	0	0	0	2b	muc	+	-	1	1	2
79	high	m	56	3	1	0	0	0	0	2b	tub1-pap	+	+	2	1	3

80	low	m	58	4a	3	0	0	1	1	4	sig	+	+	1	1	2
81	low	f	70	4b	1	0	0	1	1	4	por2	+	+	1	1	2
82	high	m	82	2	0	0	0	0	0	1b	sig	+	+	1	2	3
83	low	m	84	4a	2	0	0	0	0	3b	por1	+	+	1	1	2
84	high	f	76	4a	1	0	0	0	0	3a	por2	+	+	1	2	3
85	low	f	49	4a	3	0	0	0	0	4	por1	+	+	1	1	2
86	low	m	63	3	1	0	0	0	0	2b	por	+	+	1	1	2
87	high	f	72	2	0	0	0	0	0	1b	por2,sig	+	-	1	2	3
88	low	m	82	3	0	0	0	0	0	2a	tub2	+	+	1	1	2
89	high	m	83	3	0	0	0	0	0	2a	tub2	+	+	2	2	4
90	high	f	80	4a	2	0	1	1	0	4	por2	+	+	2	1	3
92	low	f	58	4a	0	0	1	1	1	4	por2	+	+	1	1	2
93	low	m	60	4a	2	0	0	1	1	4	tub2,por2	+	+	0	0	0
94	low	m	60	4a	0	0	0	1	1	4	tub2	+	-	1	1	2
95	high	m	57	2	1	0	0	0	0	2a	por2-sig	+	-	1	2	3
97	low	m	78	4b	2	1	0	1	0	4	tub2	+	+	0	0	0
98	high	m	61	3	0	0	0	0	0	2a	muc	+	+	1	2	3
99	high	m	70	4a	1	0	0	1	1	4	tub2	+	+	2	1	3
100	low	m	78	3	0	0	0	0	0	2a	por2	+	-	1	1	2
101	low	m	70	2	1	0	0	0	0	2a	tub2	+	+	1	1	2
102	high	f	55	4a	2	0	1	1	1	4	sig	+	+	2	1	3
103	high	m	64	4a	0	0	0	0	0	2b	por2	-	-	1	2	3
104	high	f	53	4a	2	0	0	1	1	4	sig	+	+	1	3	4
105	low	m	86	2	0	0	0	0	0	1b	pap	+	+	1	1	2
106	low	m	70	2	1	0	0	0	0	2a	pap	+	-	1	1	2
107	high	m	82	3	2	0	0	1	0	4	tub2	-	+	1	2	3
108	low	m	69	3	1	0	0	0	0	2b	por2	+	+	1	1	2
110	high	f	67	1b	1	0	0	0	0	1b	tub1	+	-	2	1	3
111	high	m	55	2	0	0	0	0	0	1b	por1	+	+	1	2	3
112	low	m	72	2	0	0	0	0	0	1b	tub2	+	+	0	0	0
113	low	f	81	3	0	0	0	0	0	2a	tub2	-	+	0	0	0
114	low	f	76	3	0	0	0	0	0	2a	por1	+	+	1	1	2
115	high	m	65	3	1	0	0	1	1	4	tub2	+	+	2	1	3
116	low	f	70	4a	1	0	0	0	0	3a	tub1	+	-	1	1	2

Gender, m: male, f: female. pT, pathological tumor depth; T1a: tumor invades lamina propria or muscularis mucosae, T1b: tumor invades submucosa, T2: tumor invades muscularis propria, T3: tumor invades subserosa, T4a: tumor perforates serosa, T4b: tumor invades adjacent structures. pN, pathological lymph node metastasis; N0: no regional lymph node metastasis, N1: metastasis in 1 to 2 regional lymph nodes, N2: metastasis in 3 to 6 regional

lymph nodes, N3: metastasis in 7 or more regional lymph nodes. H, liver metastasis; H0: no liver metastasis, H1: liver metastasis. P, peritoneal dissemination; P0: no peritoneal dissemination, P1 peritoneal dissemination. M, distant metastasis; M0: no distant metastasis, M1: distant metastasis. CY, peritoneal lavage cytology; CY0: no peritoneal lavage cytology, CY1: peritoneal lavage cytology. pStage, pathological stage (TNM stage); StageIA: T1N0M0, StageIB: T1N1M0 or T2N0M0, StageIIA: T1N2M0 or T2N1M0 or T3N0M0, StageIIB: T1N3aM0 or T2N2M0 or T3N1M0 or T4aN0M0, StageIIIA: T2N3aM0 or T3N2M0 or T4aN1M0 or T4aN2M0 or T4bN0M0, StageIIIB: T1N3bM0 or T2N3bM0 or T3N3aM0 or T4aN3aM0 or T4bN1M0 or T4bN2M0, StageIIIC: T3N3bM0 or T4aN3bM0 or T4bN3aM0 or T4bN3bM0, StageIV: anyTanyNM1. Histology, pap: papillary adenocarcinoma, tub1: well differentiated tubular adenocarcinoma, tub2: moderately differentiated tubular adenocarcinoma, por1: solid type poorly differentiated adenocarcinoma, por2: non-solid type poorly differentiated adenocarcinoma, sig: signet-ring cell carcinoma, muc: mucinous adenocarcinoma. ly, lymphatic invasion; ly (-): no lymphatic invasion, ly (+):lymphatic invasion. v, venous invasion; v (-): no venous invasion, v (+): venous invasion.

DS, Distribution of CLIC3 staining in the section; 0: 0% of total area, 1: 1-50%, 2: 51-100%. IS, Intensity of CLIC3 staining; 0: absent, 1: weak, 2: moderate, 3: strong. sum, sum of DS and IS.
