# Supplementary figures and images for: Pathophysiological properties of CLIC3 chloride channel in human gastric cancer cells
Source: J Physiol Sci. 2020 Feb 17;70:15. doi: 10.1186/s12576-020-00740-7 (PMC7026216; doi:10.1186/s12576-020-00740-7)

Additional file 2: Fig. S1.

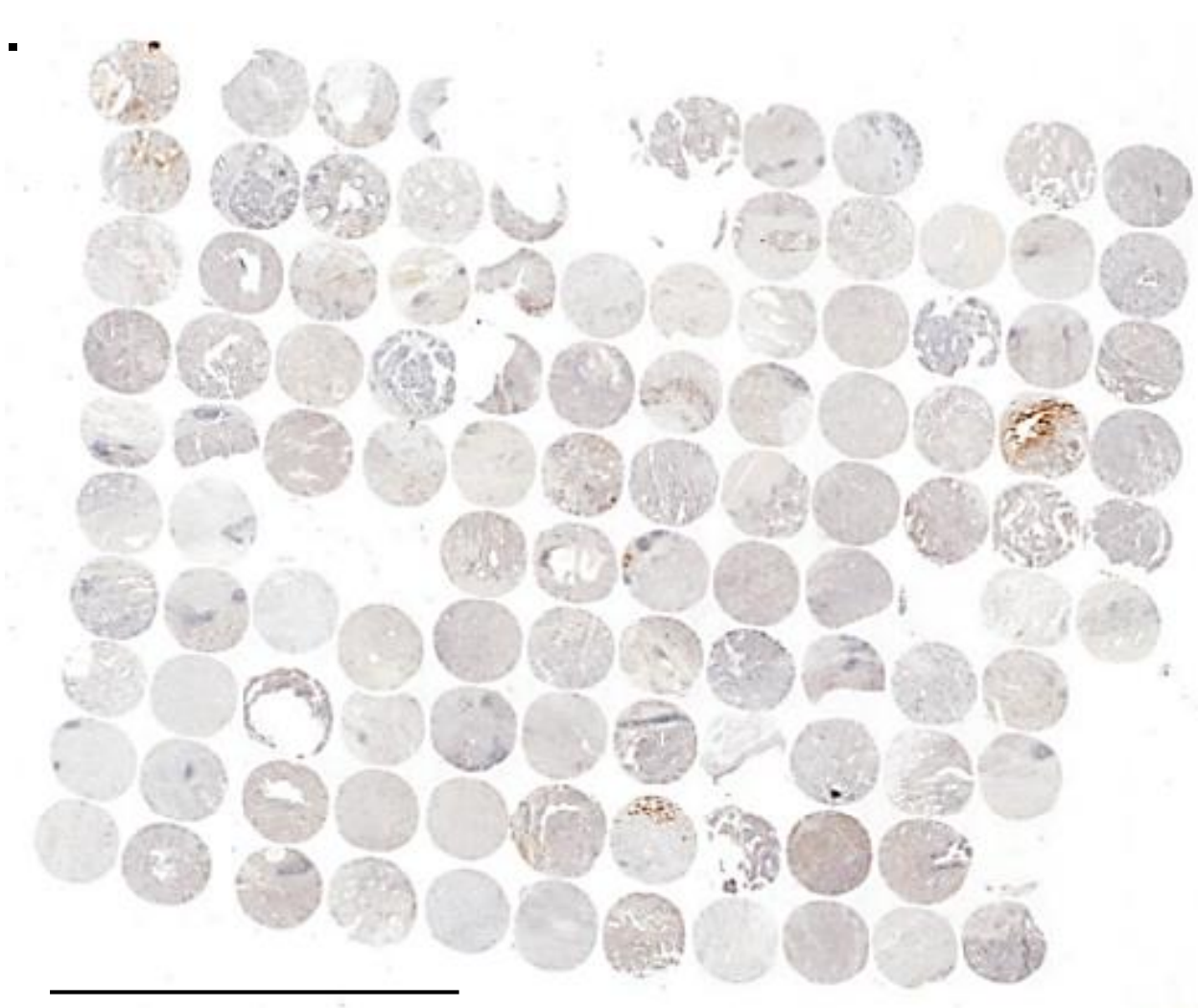

Specimen numbers

|    |    |    |    |    |    |    |    |    |     |     |     |
|----|----|----|----|----|----|----|----|----|-----|-----|-----|
| 1  | 11 | 21 | 31 |    |    | 61 | 71 | 81 |     | 101 | 111 |
| 2  | 12 | 22 | 32 | 42 |    |    | 72 | 82 | 92  | 102 | 112 |
| 3  | 13 | 23 | 33 | 43 | 53 | 63 | 73 | 83 | 93  | 103 | 113 |
| 4  | 14 | 24 | 34 | 44 | 54 | 64 | 74 | 84 | 94  | 104 | 114 |
| 5  | 15 | 25 | 35 | 45 | 55 | 65 | 75 | 85 | 95  | 105 | 115 |
| 6  | 16 |    |    | 46 | 56 | 66 | 76 | 86 |     | 106 | 116 |
| 7  | 17 | 27 | 37 | 47 | 57 | 67 | 77 | 87 | 97  | 107 |     |
| 8  | 18 | 28 | 38 | 48 | 58 | 68 | 78 | 88 | 98  | 108 |     |
| 9  | 19 | 29 | 39 | 49 | 59 | 69 | 79 | 89 | 99  |     |     |
| 10 | 20 | 30 | 40 | 50 | 60 | 70 | 80 | 90 | 100 | 110 |     |

Supplement: Supplementary file 2 — Additional file 2: Fig. S1. Enlarged image of tissue microarray analysis (Fig. 1a) and the specimen numbers. [file 12576_2020_740_MOESM2_ESM.pdf]

**Additional file 3: Fig. S2. (a)**

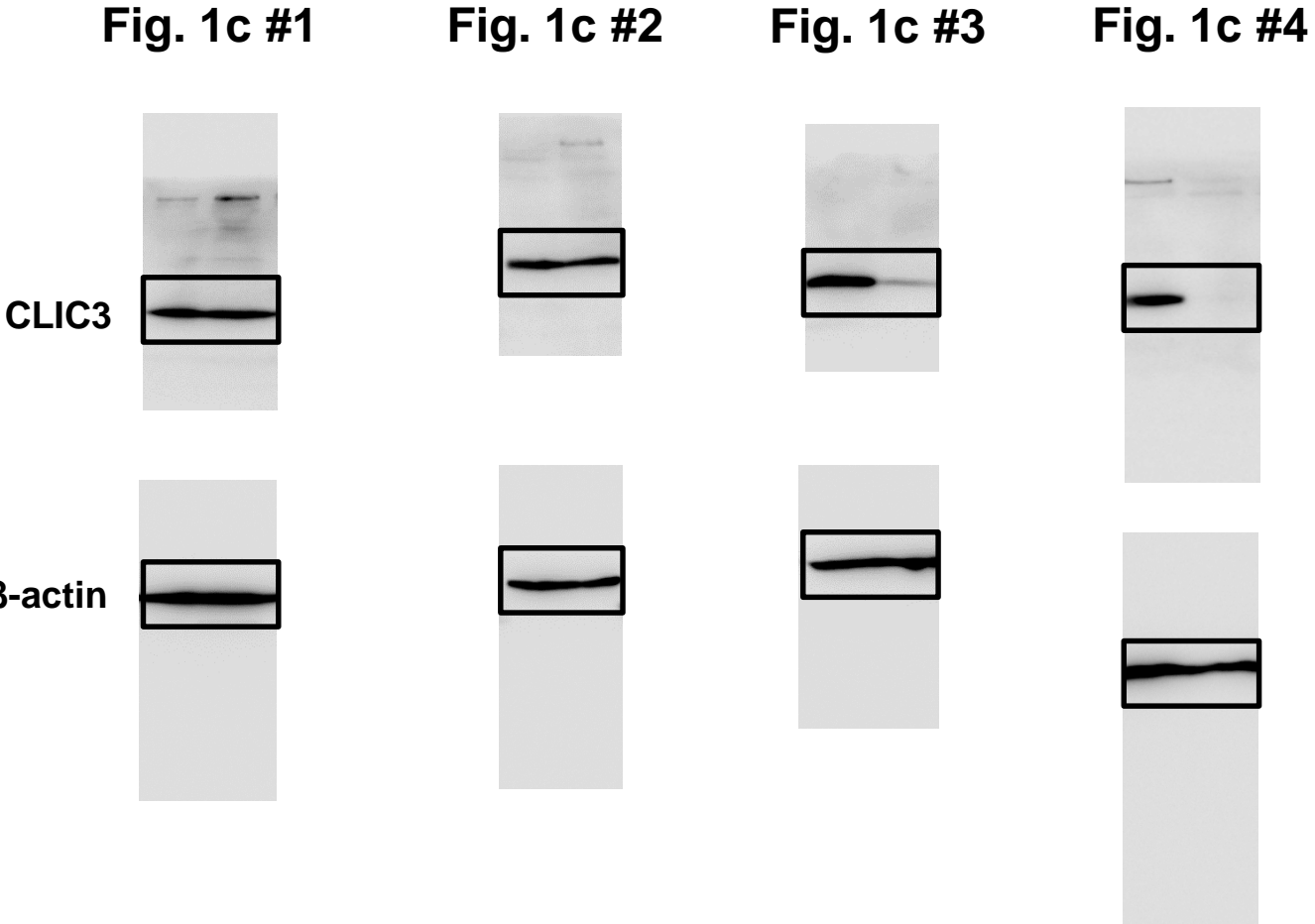

Additional file 3: Fig. S2. (b)

Fig. 1d

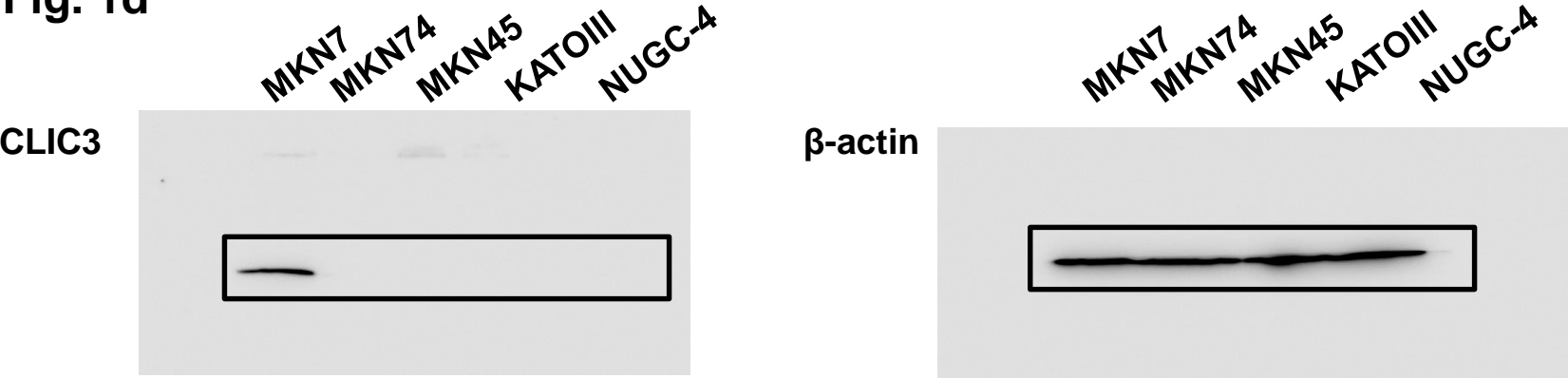

Fig. 3a

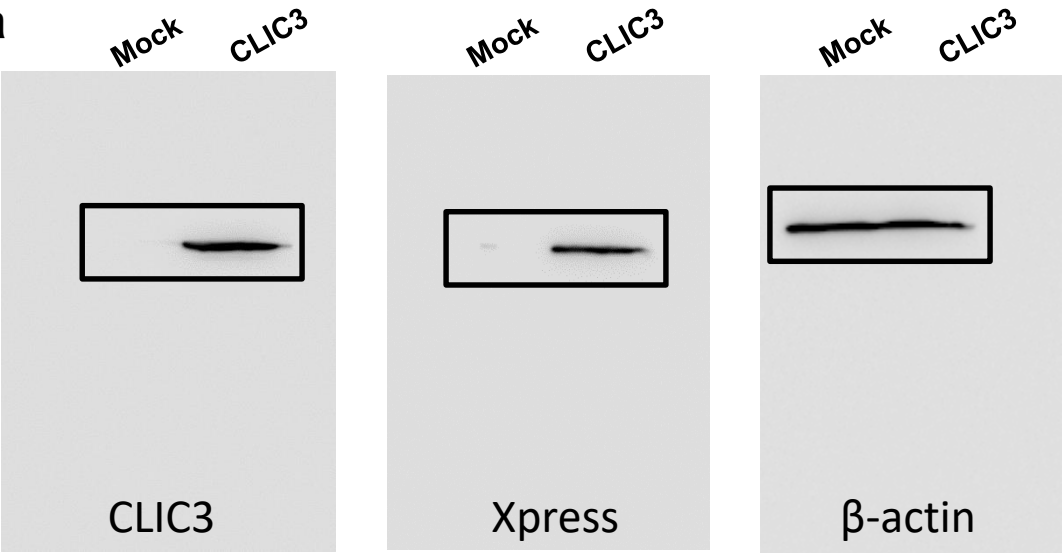

Fig. 4b

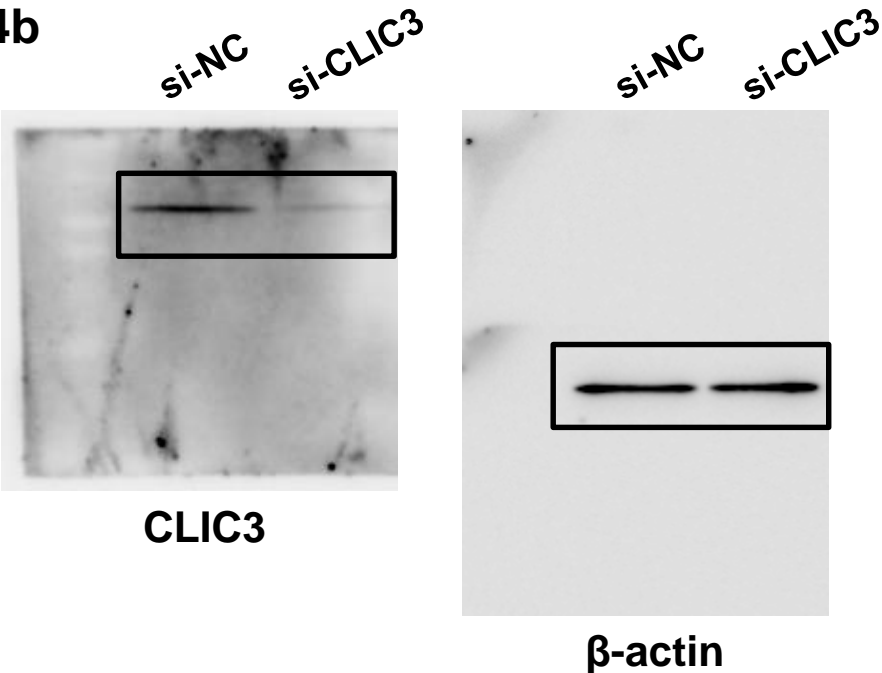

Fig. 5a

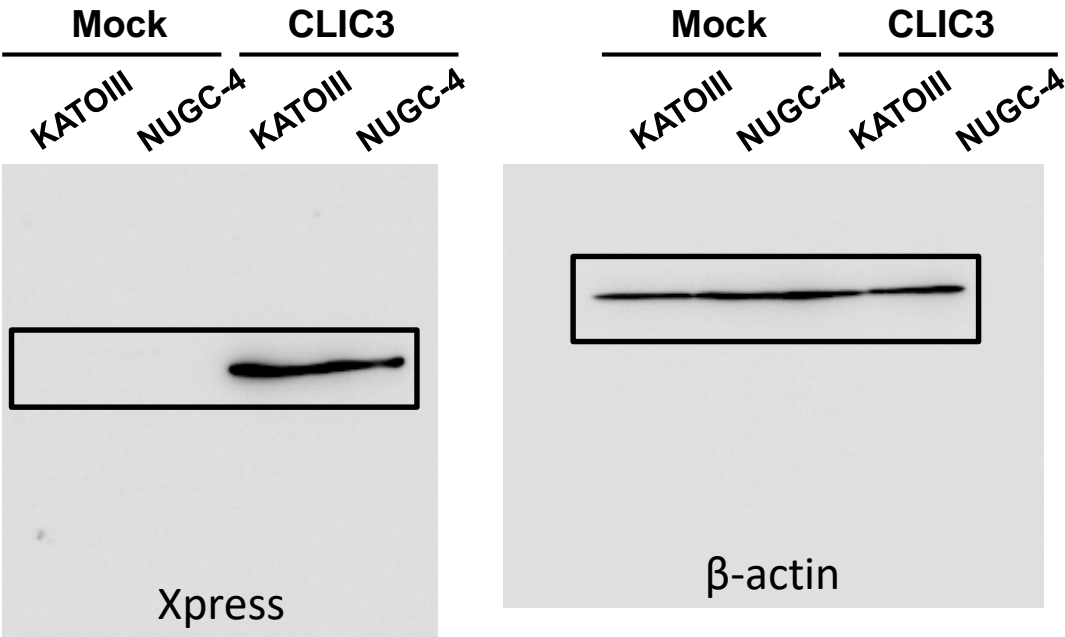

Supplement: Supplementary file 3 — Additional file 3: Fig. S2. Full images of Western blotting shown in Fig. 1c (a), Figs. 1d and 3a (b) and Figs. 4b and 5a (c). [file 12576_2020_740_MOESM3_ESM.pdf]

Additional file 4: Fig. S3.

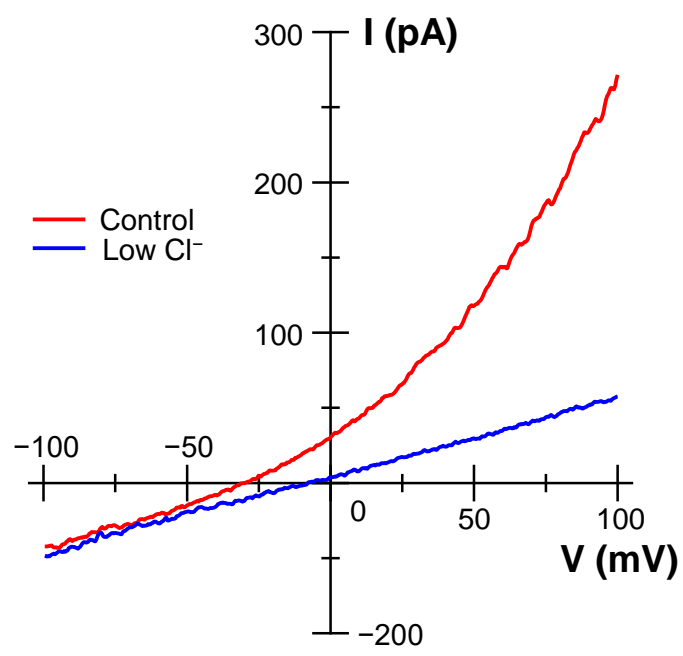

Supplement: Supplementary file 4 — Additional file 4: Fig. S3. Representative current-voltage relationships of CLIC3 currents recorded with ramp pulses (100 ms) from − 100 mV to + 100 mV in the CLIC3-expressing HEK293T cells exposed to standard bathing solution (control: red) and the low Cl− bathing solution (blue). [file 12576_2020_740_MOESM4_ESM.pdf]
